# Rostro-Caudal Organization of Connectivity between Cingulate Motor Areas and Lateral Frontal Regions

**DOI:** 10.3389/fnins.2017.00753

**Published:** 2018-01-11

**Authors:** Kep Kee Loh, Fadila Hadj-Bouziane, Michael Petrides, Emmanuel Procyk, Céline Amiez

**Affiliations:** ^1^Univ Lyon, Université Claude Bernard Lyon 1, Institut National de la Santé Et de la Recherche Médicale, Stem Cell and Brain Research Institute U1208, Bron, France; ^2^Institut National de la Santé Et de la Recherche Médicale, U1028, Centre National de la Recherche Scientifique UMR5292, Lyon Neuroscience Research Center, ImpAct Team - University UCBL Lyon 1, Lyon, France; ^3^Department of Neurology and Neurosurgery, Montreal Neurological Institute, Montreal, QC, Canada

**Keywords:** frontal cortex, functional gradient, resting-state fMRI, task-related fMRI, humans, cingulate motor areas

## Abstract

According to contemporary views, the lateral frontal cortex is organized along a rostro-caudal functional axis with increasingly complex cognitive/behavioral control implemented rostrally, and increasingly detailed motor control implemented caudally. Whether the medial frontal cortex follows the same organization remains to be elucidated. To address this issue, the functional connectivity of the 3 cingulate motor areas (CMAs) in the human brain with the lateral frontal cortex was investigated. First, the CMAs and their representations of hand, tongue, and eye movements were mapped via task-related functional magnetic resonance imaging (fMRI). Second, using resting-state fMRI, their functional connectivity with lateral prefrontal and lateral motor cortical regions of interest (ROIs) were examined. Importantly, the above analyses were conducted at the single-subject level to account for variability in individual cingulate morphology. The results demonstrated a rostro-caudal functional organization of the CMAs in the human brain that parallels that in the lateral frontal cortex: the rostral CMA has stronger functional connectivity with prefrontal regions and weaker connectivity with motor regions; conversely, the more caudal CMAs have weaker prefrontal and stronger motor connectivity. Connectivity patterns of the hand, tongue and eye representations within the CMAs are consistent with that of their parent CMAs. The parallel rostral-to-caudal functional organization observed in the medial and lateral frontal cortex could likely contribute to different hierarchies of cognitive-motor control.

## Introduction

Prevailing theories about the functional organization of the frontal lobe suggest that the lateral frontal cortex is organized along a rostral-to-caudal axis of behavioral/cognitive control where higher level cognitive processing is implemented rostrally and motor control processing, caudally (Petrides, 2005a,b; Koechlin and Summerfield, 2007; Badre and D'Esposito, 2009). Whether the medial frontal cortex, which is strongly connected with the lateral frontal cortex (Dum and Strick, 1991; Bates and Goldman-Rakic, 1993; Lu et al., 1994), is organized along a similar axis remains to be demonstrated. A recent study has provided support for this hypothesis by showing that, along a rostral-caudal axis, progressively rostral medial frontal regions are involved in monitoring the reliability of more complex behavioral rules maintained in progressively rostral lateral frontal regions, and to seek out new alternatives as current rules become unreliable (Domenech and Koechlin, 2015). Within the medial frontal cortex, the rostral cingulate motor region appears to play a key role in frontal medio-lateral interactions during complex adaptive decision-making (Procyk et al., 2016). In this context, the goal of the present article is to provide new insights into the organization of the human cingulate motor cortex, by examining its functional connectivity with the lateral frontal cortex.

Anatomical investigations of the monkey cingulate cortex have demonstrated at least 3 cingulate motor areas (CMAs): CMAr, CMAd, and CMAv based on their relative positions in the rostral, dorsal and ventral parts of the cingulate sulcus, respectively (He et al., 1995). The two posterior areas have sometimes been regarded as a single CMAc caudal region. Anterior and posterior CMAs have also been labeled as M3 and M4 by Morecraft et al. (1996). CMAs constitute the main sites of cingulate connections with the precentral motor and premotor cortex, and the spinal cord (Dum and Strick, 1991; Morecraft and Van Hoesen, 1992). The CMAs also display reciprocal connections with the prefrontal cortex (Bates and Goldman-Rakic, 1993; Morecraft and Van Hoesen, 1993; Picard and Strick, 1996). Notably, the most rostral region, CMAr, in contrast with the more caudal CMAd and CMAv, has denser connections with the pre-SMA, orbital, medial and lateral prefrontal cortex, weaker connections with the dorsal premotor and primary motor cortex, and fewer but a more complex pattern of cortico-spinal projections (Dum and Strick, 1991; Luppino et al., 1993; He et al., 1995; Hatanaka et al., 2003; Morecraft et al., 2004, 2012; Petrides and Pandya, 2006). These findings suggest that, along a rostro-caudal axis, CMAs show graded relationships with prefrontal (decreasing connectivity) and motor regions (increasing connectivity). These connectivity trends corroborate that the anterior CMA is more implicated in higher-order cognitive functions in association with the prefrontal cortex and the posterior CMAs in motor functions associated with the motor, premotor cortex and spinal cord.

In the human brain, the cingulate cortex contains three premotor areas—also known as the cingulate motor zones: the rostral anterior cingulate zone (RCZa) and the rostral posterior cingulate zone (RCZp) in the midcingulate cortex (MCC), and the caudal cingulate zone (CCZ) in the posterior cingulate cortex (Picard and Strick, 1996, 2001; Beckmann et al., 2009; Amiez and Petrides, 2014). Neuroimaging experiments suggest that these zones are somatotopically organized, with RCZa and RCZp containing both limb and face motor representations, and the CCZ containing only limb motor representations (Amiez and Petrides, 2014). Several studies and meta-analyses have emphasized the anatomo-functional correspondence between RCZa in humans and CMAr in monkeys (Shackman et al., 2011; Amiez and Petrides, 2014; Procyk et al., 2016) and suggested possible correspondences between the human RCZp and CCZ with the monkey posterior cingulate motor regions (Amiez and Petrides, 2014). At this point, an important question remains unresolved: Is the rostral-to-caudal organization of decreasing prefrontal and increasing motor cortex connectivity observed in the monkey CMAs also present in the human cingulate zones? This question is critical not only in establishing the anatomo-functional homologies between monkey CMAs and human cingulate zones but also in shedding light on the functional interactions between the human medial and lateral frontal cortical areas.

Here, we aim to describe the connectivity profiles of the 3 cingulate zones in the human brain (that we group under the generic term CMAs) and crucially, to test if they reflect a rostro-caudal cognitive-to-motor functional organization as in the lateral frontal cortex. To this end, we combined task-related fMRI, to map the CMAs and their motor representations, with resting-state fMRI, to examine their functional connectivity with various lateral frontal cortical regions situated along a rostro-caudal axis. Importantly, these analyses were conducted on a subject-by-subject basis, since this is the only way to dissociate the various motor representations in the RCZa and RCZp. Indeed, Amiez and Petrides (2014) have demonstrated that the relative locations of motor map subdivisions in RCZa and RCZp vary according to individual sulcal morphology: when a paracingulate sulcus (PCGS) is present (in about 70% of subjects in at least one hemisphere), the face motor representations of the RCZa and RCZp are located in the PCGS, whereas the limb motor representations are located in the cingulate sulcus. Conversely, when a PCGS is absent, all motor representations of each CMA are located in the cingulate sulcus.

The present study demonstrates the existence of a rostro-caudal functional organization of the CMAs in the human brain based on their differential coupling with lateral frontal brain regions along a rostro-caudal axis. The most anterior CMA has stronger functional connectivity with the rostral prefrontal areas, whereas posterior CMAs, exhibiting an opposite pattern of connectivity, have stronger connectivity with the (more caudal) motor regions. We discuss the implications of these findings in the context of the involvement of frontal medio-lateral brain networks in behavioral control.

## Method

### Subjects

For this study, 23 healthy, right-handed native French speakers were recruited, but two subjects had to be excluded because of claustrophobia. Thus, 21 subjects (12 males; mean age of all subjects 26.0, SD = 3.94) were included in the final analysis. The study was carried out in accordance with the recommendations of the Code de la Santé Publique and was approved by the “Agence Nationale de Sécurité des médicaments et des produits de santé (ansm)” and the “Comité de Protection des Personnes (CPP) Sud-Est III” (N° EudraCT: 2014-A01125-42). It also received a Clinical Trial Number (NCT03124173, see https://clinicaltrials.gov). All subjects gave written informed consent in accordance with the Declaration of Helsinki.

### Tasks

**Figure 1:**
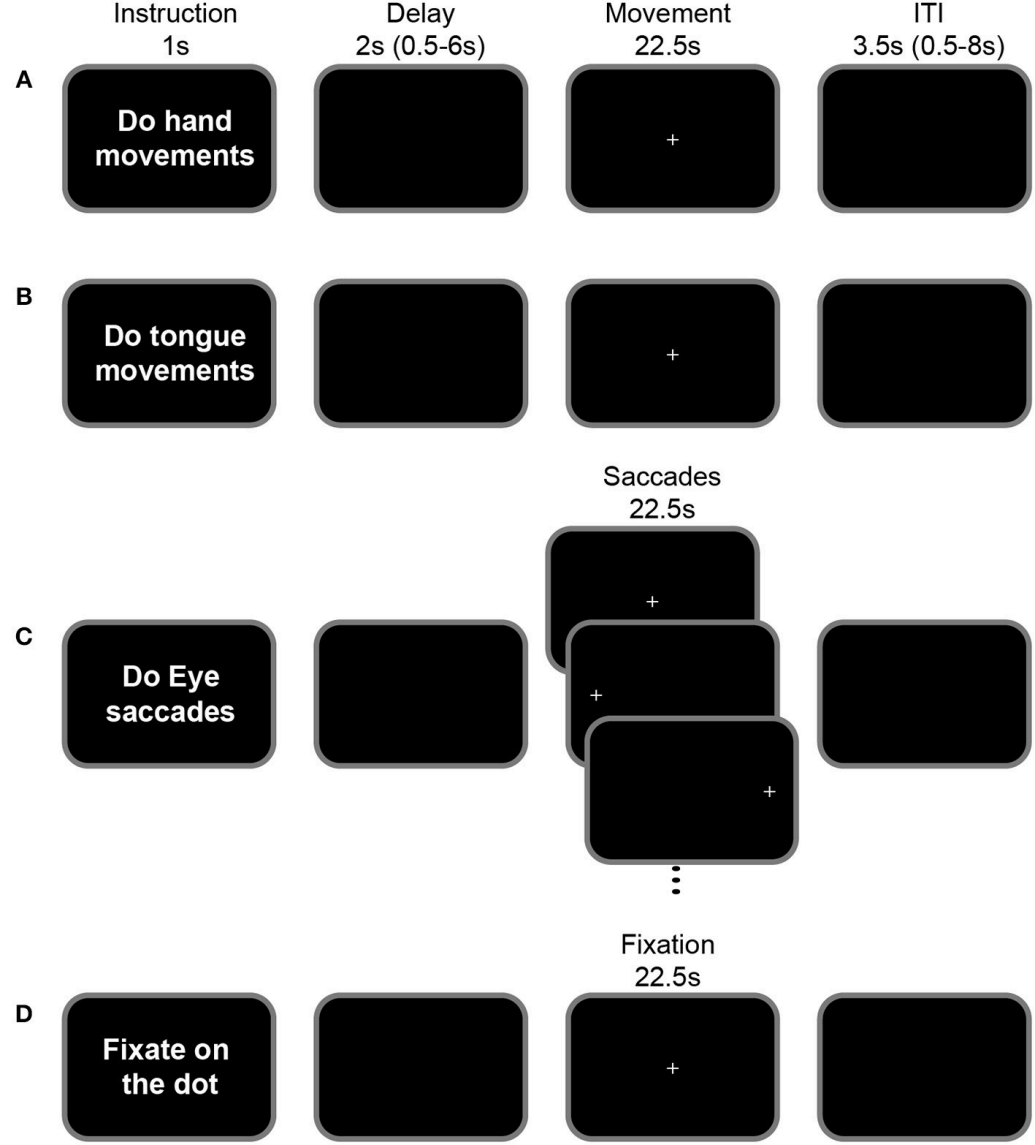
Motor-mapping fMRI task. The general task structure was identical across hand **(A)**, tongue **(B)**, saccadic eye **(C)** movements and ocular fixation blocks **(D)**. Each task began with an instruction screen (1 s) followed by a variable delay (0.5–6.0 s, mean = 2 s). The appearance of a central cross indicated the start of the movement period (22.5 s) during which the instructed action is performed: In the hand movement condition **(A)**, subjects moved their right hand up and down, while keeping their arm fixed beside their body on the scanner bed. In the tongue condition **(B)**, subjects moved their tongue (clockwise circular movements), with their mouth closed. In the eye saccade condition **(C)**, subjects shifted their gaze according to the position of the presented cross which alternated between the left, central and right positions (750 ms at each position). In the fixation condition **(D)**, subjects fixated on a central cross. The inter-trial interval varied between 0.5 and 8 s (mean = 3.5 s).

To map the different motor areas in the cingulate cortex, we adopted the protocol described in Amiez and Petrides (2014). Subjects performed right hand, tongue, and saccadic eye movements during fMRI scanning (see Figure 1). A short sentence was presented on the screen for 1 s indicating to the subject the type of movement that they would have to perform in the trial (instruction period). After a jittered delay varying from 0.5 to 6.0 s (average = 2 s), a fixation point was presented for 22.5 s. The occurrence of this fixation point was the signal to perform the movement indicated in the preceding instruction period while maintaining eye fixation during the performance of the required movement. The disappearance of the fixation point 22.5 s later instructed the subject to stop performing the movement and an inter-trial interval (ITI) followed. The short instruction sentence “Do hand movements” informed the subject to move the right hand up and down while keeping the arm and wrist on the scanner bed and the fingers straight (Figure 1A) and the instruction “Do tongue movements” informed the subject to rotate the tongue clockwise while keeping the mouth closed (Figure 1B). The protocol was slightly different for the assessment of the eye fields since the subjects were asked to perform saccadic eye movements. In this case, the sentence “Do eye saccades” indicated to the subjects that, after the 0.5–6 s jittered delay (average = 2 s), they would have to perform a saccade to follow a dot presented in 1 of 3 possible locations on the screen (left, middle, or right), for 22.5 s. Each dot appeared for 750 ms at each location (Figure 1C). This protocol has been described in detail in Amiez et al. (2006) and Amiez and Petrides (2009, 2014). Finally, in the control condition, the instruction sentence was “Fixate the central cross” and the subject had to maintain an ocular fixation on the dot presented in the center of the screen during 22.5 s (Figure 1D). After the movements/ocular fixation, an inter-trial interval 0.5–8.0 s (average = 3.5 s) was presented. The presentation of the stimuli was controlled via Presentation software (Neurobehavioral systems). The subjects viewed the stimuli in the scanner via an LCD projector with a mirror system.

### MRI acquisition

Scanning was performed on a 3T Siemens Magnetom Prisma MRI Scanner (Siemens Healthcare, Erlangen, Germany). To minimize movements during the motor tasks, the head of the subject was tightly cushioned throughout the acquisition of fMRI data. The functional MRI data (T2^*^-weighted gradient echo planar EPI images, 40 oblique slices, voxel resolution = 2.7 × 2.7 × 2.7 mm, TR = 2.2 s, TE = 30.0 s, flip angle = 90°) from the fixation and motor mapping tasks were acquired over two MRI sessions. In the first session, each run consisted of one fixation and one tongue mapping block. In the second session, subjects performed one fixation and two mapping blocks (hand and saccades) in each run. Note that in both sessions, subjects had additionally performed a second cognitive task, as part of a separate protocol, after the fixation and motor mapping blocks. However, in the present article, we analyze only the data from the fixation and motor-mapping blocks. In both sessions, subjects performed 4–6 acquisition runs. In the first session, we acquired in each subject, 160–240 TRs of data corresponding to the fixation (22.5 s/2.2 ^*^ 4–6 runs = 80–120 TRs) and tongue movement trials (22.5 s/2.2 ^*^ 4–6 runs = 80–120 TRs). In the second session, we acquired in each subject, 240–360 TRs of data from the fixation, saccadic eye and hand movement trials. High resolution T1-weighted anatomical scans (MPRAGE, 0.9 mm^3^ isotropic voxels, 192 slices, TR = 3.5 s, TE = 2.67 s) were also acquired during one of the experimental sessions.

The resting-state functional MRI scan was performed in one experimental session after all the task runs. Three-hundred EPI images were acquired over 11 min with identical scanning parameters as the task-based functional scans. During this acquisition, subjects were instructed to keep still and maintain fixation on a white dot presented on the center of the screen while blinking normally. Photoplethysmography (PPG) data were acquired via an infrared pulse oximeter (Siemens Healthcare, Erlangen, Germany) attached to the subject's left index finger. A Biopac MP150 system (Biopac Systems Inc, Goleta, CA) was used to acquire simultaneously and synchronize the PPG signal from the oximeter with the TTL pulses from the MRI scanner. Eye-tracking was achieved by using monocular corneal reflection and pupil tracking via an SR Research Eyelink 1,000 long-range MRI Eyetracker (SR Research, Ontario, Canada) sampling at 1,000 Hz. For synchronizing fMRI and eyetracking data, TTL pulses from the scanner were delivered into the computer controlling the eye movements via a parallel port connection.

### MRI data analysis

#### Task-related fMRI

Functional data from the mapping/fixation task runs were preprocessed via Statistical Parametric Mapping software (SPM12; Wellcome Department of Cognitive Neurology, UCL, UK; http://www.fil.ion.ucl.ac.uk/spm) and Matlab 16a (http://www.mathworks.com). The first 5 volumes of each run were removed to allow for *T*_1_ equilibrium effects. We applied a slice-timing correction using the time center of the volume as reference. Then, head motion correction was applied using rigid-body realignment. These realignment parameters were used as covariates during the statistical analysis to model out potential nonlinear head motion artifacts. Functional and morphological images were spatially normalized into standard MNI space using SPM's default templates. Functional data were finally smoothed using a 6-mm full-width half maximum Gaussian kernel (Friston et al., 1995a,b,c). A 128-s temporal “high-pass filter” regressor set was included in the design matrix to exclude low-frequency confounds.

Each task trial was modeled with impulse regressors at the time of the presentation of the fixation point that initiated the performance of the hand, tongue, and saccadic eye movements, as well as ocular fixation. These regressors were then convolved with the canonical hemodynamic response function and entered into a general linear model (GLM) of each subject's fMRI data. The 6 scan-to-scan motion parameters produced during realignment were included as additional regressors in the GLM to account for residual effects of subject movement. Statistical significance in the resulting single-subject contrast images was assessed with a *p*_*uncorrected*_ < 0.001 voxel-wise threshold.

#### Resting state fMRI

The preprocessing of resting-state scans was also performed with SPM 12. The first 5 volumes of each run were removed to allow for *T*_1_ equilibrium effects. We then applied a slice-timing correction using the time center of the volume as reference. The head motion correction was applied using rigid-body realignment and, then, using the AFNI software (Cox, 1996), a temporal filtering was applied to extract the spontaneous slowly fluctuating brain activity (0.01–0.1 Hz). Finally, a linear regression was used to remove nuisance variables (the six parameter estimates for head motion, the cerebrospinal fluid and white matter signals from the SPM segmentation, the number of heart pulses per TR, and the mean pupil size per TR). Note that a customized Matlab program was used to detect and count the number of peaks in the PPG signal in each TR window. Automatic peak detection was performed using the “findpeaks” function from the Signal Processing Toolbox (https://www.mathworks.com). Raw pupil diameter data were extracted from the Eyelink datafile and subsequently averaged in each TR window via Matlab. Finally, a spatial smoothing with a 6-mm FWHM Gaussian kernel was applied to the output of the regression.

The main goal of the resting-state fMRI analyses was to investigate, on a subject-by-subject basis, the differential functional connectivity associated with the various CMAs as well as their individual motor representations (i.e., tongue, eye, and hand). Specifically, our analyses were focused on the left intra-hemispheric connectivity of the CMAs (and their motor representations) with the lateral prefrontal and motor cortical areas, as well as with connectivity between the CMAs. For this analysis, a total of 15 locations in the left hemisphere of each subject were identified: in the CMAs (8 seeds), prefrontal cortex (5 ROIs), and motor cortex (3 ROIs) (see below and **Figure 3**).

#### Seed selection in CMAs

**Figure 2:**
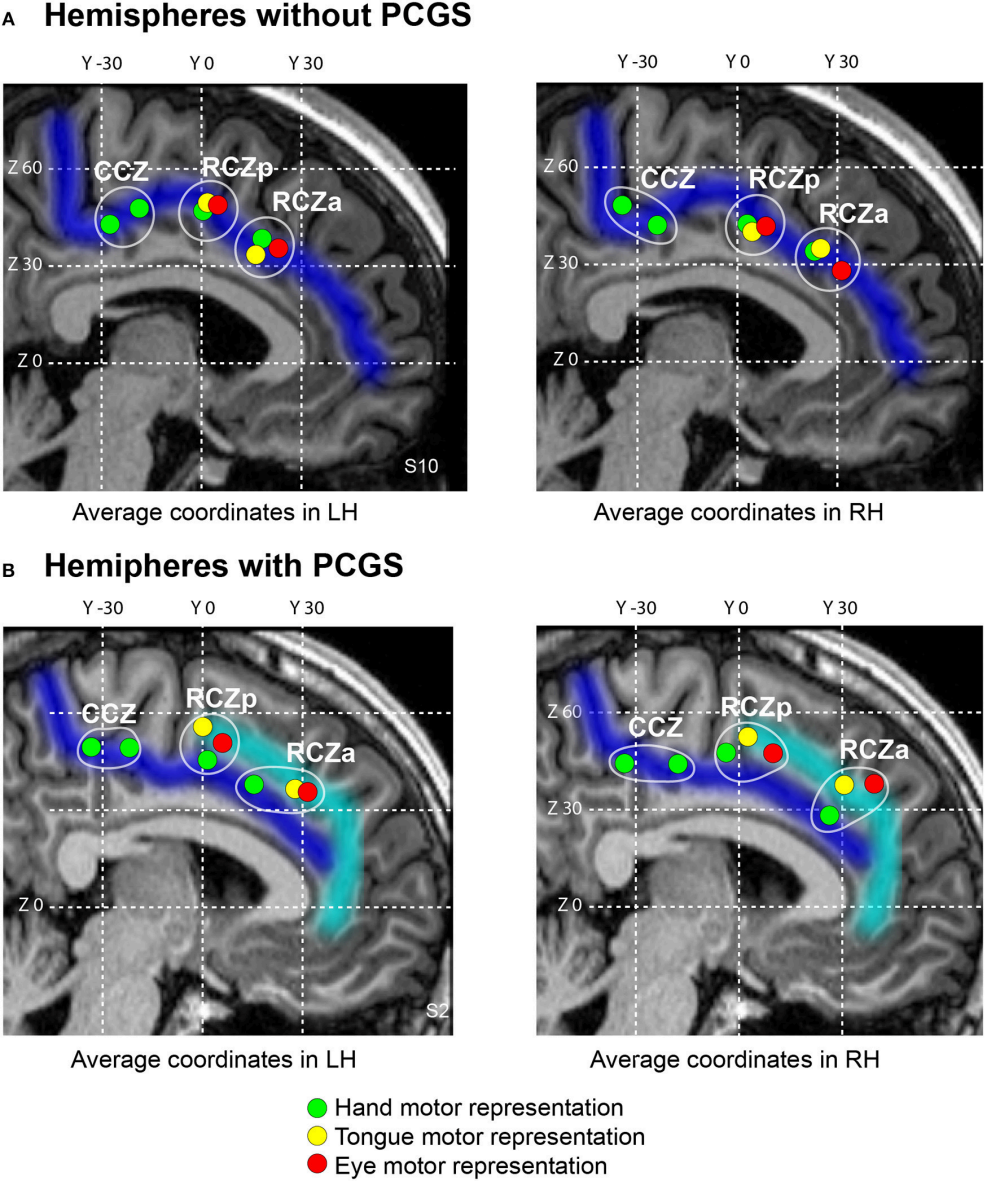
Somatotopic organization of the human cingulate motor areas. The average locations of individual subject's peak activations associated with the performance of hand (green), eye saccadic (red) and tongue (yellow) movements plotted on a typical hemisphere with cingulate sulcus (**A**, Subject 10) and paracingulate sulcus (**B**, Subject 2). In both the hemispheres without **(A)** and with **(B)** paracingulate sulcus, three anterior-to-posterior clusters of activations can be observed with the two anterior-most clusters (RCZa and RCZp) containing all hand, eye and tongue representations, and the posteriormost cluster (CCZ) which contains two hand representations. The CGS and PCGS are marked in dark blue and cyan respectively. The antero-posterior (y) and dorso-ventral (z) coordinates in the MNI standard stereotaxic space are indicated as white grid lines. cgs, cingulate sulcus; pcgs, paracingulate sulcus; RCZa, anterior rostral cingulate zone; RCZp, posterior rostral cingulate zone; CCZ, caudal cingulate zone.

**Figure 3:**
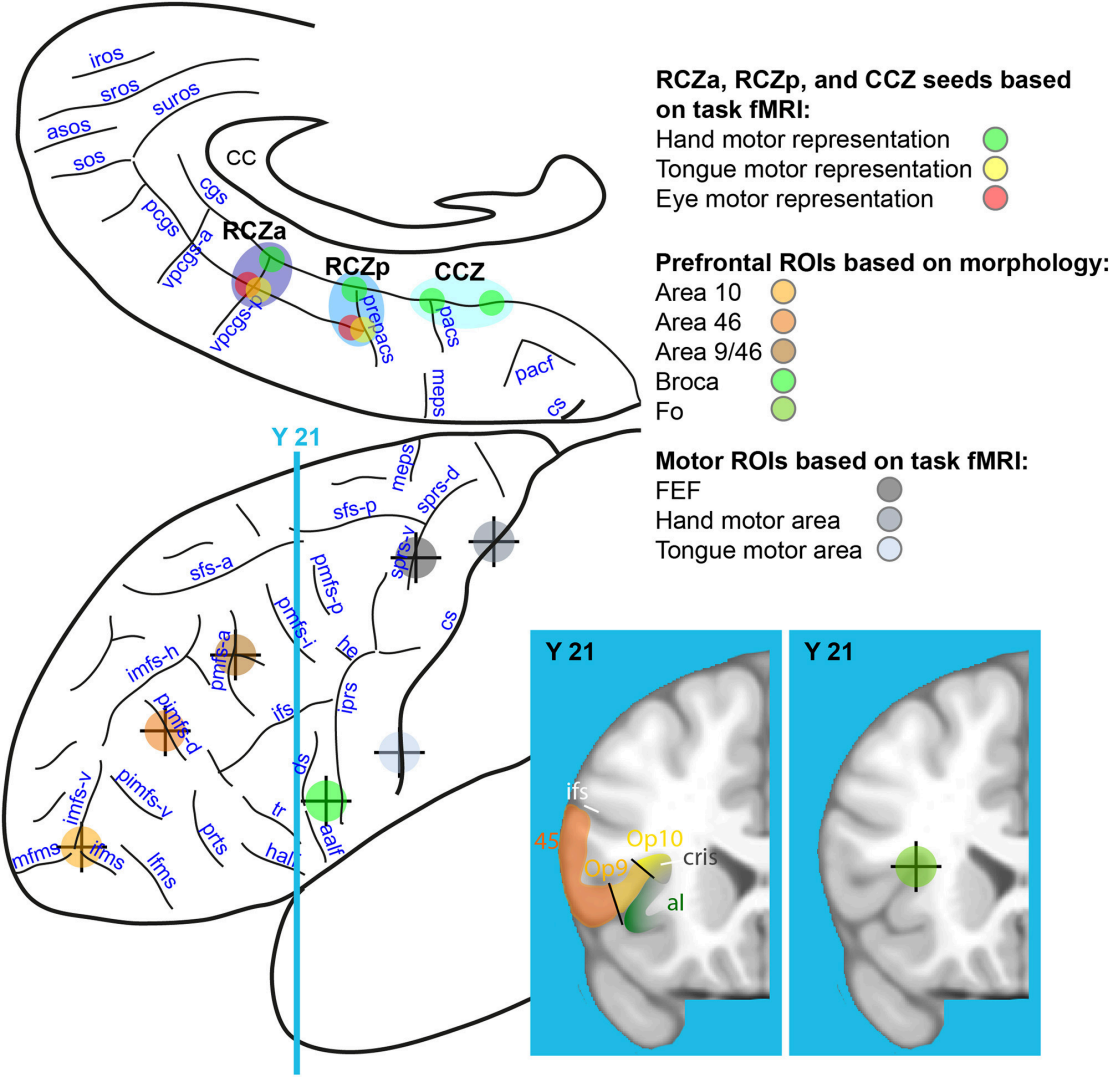
Rostro-caudal CMAs seed locations on the medial frontal cortex and ROI locations on the lateral frontal cortex. Eight seeds were identified on the basis of task-fMRI results in the medial frontal cortex: hand (in green), tongue (in yellow), and eye (in red) motor representations of the RCZa (dark blue), RCZp (medium blue), and CCZ (light blue). Seven ROIs were identified in the lateral frontal cortex along a rostral-to-caudal axis based on individual subject anatomy (4 prefrontal ROIs: Area 10, Area 46, Area 9/46 and Broca's Area) and from the motor mapping task (3 ROIs: FEF, hand and face precentral motor regions). The center of the crosses indicates the point of the sulci in the depth of which the various ROIs/seeds are located. CC, corpus callosum; cs, central sulcus; meps, medial precentral sulcus; pacf, paracentral fossa; cgs, cingulate sulcus; pcgs, paracingulate sulcus; pacs, paracentral sulcus; prepacs, pre-paracentral sulcus; vpcgs-p and vpcgs-a, posterior and anterior vertical paracingulate sulcus; suros, supra-rostral sulcus; sros and iros, superior and inferior rostral sulcus; sos, sus-orbitalis sulcus; asos, accessory sus-orbitalis sulcus; iprs, inferior precentral sulcus; he, horizontal extension; ifs, inferior frontal sulcus; sfs-a and sfs-p, anterior and posterior part of the superior frontal sulcus; imfs-h, and imfs-v, horizontal and ventral extension of the intermediate frontal sulcus; mfms, ifms, and lfms, medial, intermediate, and lateral fronto-marginalis sulcus; pimfs-v and pimfs-d, ventral and dorsal para-intermediate frontal sulcus; pmfs-a, pmfs-i, pmfs-p, anterior, intermediate, and posterior posteromedial frontal sulcus; ds, diagonalis sulcus; ts, triangularis sulcus; aalf, anterior ramus of the lateral fissure; half; horizontal anterior ramus of the lateral fissure.

For each subject, 8 CMA seed locations in the left cingulate cortex were identified based on activation peaks from the fMRI motor-mapping task. These included the hand, eye, and tongue motor representations in RCZa and RCZp, and the two hand motor representations in CCZ (see Figures 2, 3).

#### ROIs selection in motor cortical areas

For each subject, 3 ROIs within the left motor cortex were identified based on activation peaks from the fMRI motor-mapping task. These included the hand motor region (the precentral knob) within the central sulcus –M-H– (Boling et al., 1999; Amiez et al., 2006), the tongue motor region within the ventral part of the posterior part of the precentral gyrus –M-T– (Weiss et al., 2013), the frontal eye field –FEF– within the ventral branch of the superior precentral sulcus (Amiez et al., 2006). Monkey studies have shown that the CMAs are anatomically linked with the motor cortex (e.g., Dum and Strick, 1991; Procyk et al., 2016). The motor ROIs were selected to verify the connectivity profiles of the individual motor representations in the various CMAs with the motor system. Considering that the CMAs contain tongue, eye, and hand motor representations (Amiez and Petrides, 2014), we assessed the connectivity of these regions with the primary tongue area, the FEF, and the primary hand area. Note that the CMAs also contain foot motor representations but these had not been mapped in our mapping task. As such, in the present study, we did not assess CMAs' connectivity with the primary foot motor cortex (Amiez and Petrides, 2014). Figure 3 displays the location of these ROIs.

#### ROIs selection in the prefrontal cortex

For each subject, 5 ROI locations within the left prefrontal cortex were identified based on local anatomy. These included area 10 –a10– (located at the intersection between the vertical segment of the intermediate frontal sulcus, the lateral and the medial frontomarginal sulcus, see Petrides, 2014), dorsolateral prefrontal areas 46 –a46– [within the dorsal paraintermediate frontal sulcus (pimfs-d)] and 9/46 –a9/46– [within the anterior segment of the posterior middle frontal sulcus (pmfs-a), see Amiez and Petrides, 2007], area 44 –Broca– [Broca's area, in the center of the pars opercularis, i.e., between the anterior ramus of the lateral fissure (aalf) and the inferior precentral sulcus (iprs), see (Petrides, 2014)], and the frontal operculum –Fo– (intersection between the frontal operculum and the circular sulcus, see Amiez et al., 2016). These ROIs were selected because of their known frequent co-activation with the MCC in a large range of cognitive tasks (Amiez and Petrides, 2007; Amiez et al., 2012a,b, 2013). ROI locations on the lateral surface of the prefrontal cortex are shown in Figure 3. Note that the center of each of the ROIs located in the sulci was positioned about 5 mm below the surface given the chosen size of the radius sphere (i.e., 4 mm, see below).

Based on the above 15 locations, seed/ROI spheres with a 4 mm radius were generated for each subject using the AFNI software and the mean signal form these regions was extracted. Note that this radius size was selected in order to allow the separate assessment of functional connectivity of seeds located in the CGS from those located in the PCGS when present. Indeed, the PCGS and CGS are most often separated of about 8–12 mm, so a larger radius would have prevented us to assess this putative dissociation. For each subject, correlation coefficients between the different CMA seeds with the various ROIs in the prefrontal cortex, motor cortex and other CMA seeds were computed and normalized using the Fisher's r-to-z transform formula. Significant threshold at the individual subject level was *Z* = ±0.2 (*p* < 0.001) and *Z* = ±0.15 (*p* < 0.01). These normalized correlational coefficients, which corresponded to the functional connectivity strength between each CMA seed and each ROI/other CMA seed in individual subjects, were subsequently processed with R statistical software (https://www.r-project.org/) for all the following analyses.

#### Automatic clustering based on seed-ROI correlations

To compare the connectivity between the different CMAs and lateral frontal cortex, we first averaged the normalized correlation coefficients for each seed-ROI pairing across subjects. Next, Euclidean distance vectors were separately computed for the 8 CMA seeds (based on their mean connectivity values with the ROIs) and the 8 prefrontal/motor ROIs (based on their mean connectivity values with the CMA seeds). Finally, unsupervised hierarchical clustering was performed for the CMA seeds and ROIs separately. This clustering was generated using the hclust function in R which uses the complete linkage method for hierarchical clustering. This particular clustering method defines the cluster distance between two clusters to be the maximum distance between their individual components. At every stage of the clustering process, the two nearest clusters are merged into a new cluster. The process is repeated until the whole data set is agglomerated into one single cluster. This clustering method assumes a representation of data in an Euclidean space in which it is possible to summarize a collection of points by their centroid (i.e., the average of the points) (for more details, see http://www.r-tutor.com/gpu-computing/clustering/hierarchical-cluster-analysis). The outcome was used to construct dendrograms and heatmaps. To better display clusters across ROIs, values in the heatmaps were normalized (z-scored) by column. Hence values (and sign) in the heatmap do not represent actual connectivity measures.

#### Rostro-caudal frontal axis analyses

**Figure 4:**
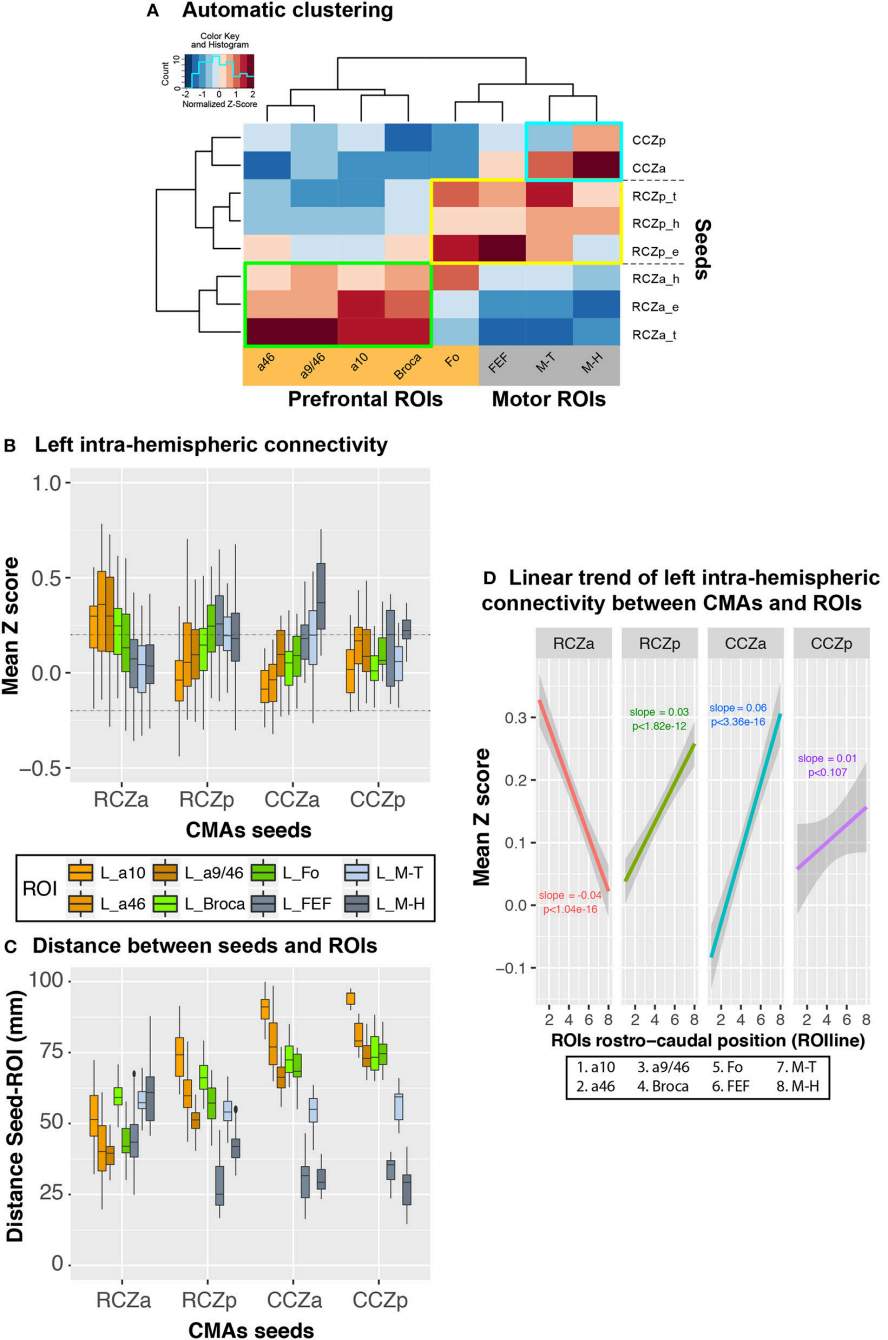
Rostro-caudal organization in left hemispheric CMA-lateral frontal cortex connectivity profiles: normalized values. **(A)** Automatic clustering of the seed-ROI connectivity values show that the various CMA seeds (left dendrogram) and lateral frontal ROIs (top dendrogram) could be identified on the basis of their interconnectivity. The heatmap shows correlation values normalized by column to better illustrate clusters. Each cell in the heatmap represents the averaged seed-ROI connectivity value (transformed correlation coefficients) across subjects and with a color scale mapped on values z-scored by column (red and blue corresponding respectively to negative and positive normalized values, not to actual connectivity correlation values). Three distinct clusters of strong correlations could be observed: (1) Green box: strong connectivity between RCZa seeds and prefrontal ROIs; (2) Blue box: strong connectivity between CCZ seeds and motor ROIs; Yellow box: RCZp seeds, which showed an intermediate pattern of connectivity between CCZ and RCZa. **(B)** Boxplots displaying the mean (±SD) z-transformed correlation between each CMA location (RCZa, RCZp, CCZa, CCZp) on the x-axis, and the various ROIs (color-coded) in all subjects. **(C)** Boxplots displaying the mean (±SD) Euclidian distance between each seed (RCZa, RCZp, CCZa, CCZp) on the x-axis, and the various ROIs (color-coded) in all subjects. **(D)** Significant linear organization in the connectivity of each CMA location with the rostral-caudal lateral frontal ROIs. The rostro-caudal organization variable (ROIline) was obtained by recoding the ROIs in terms of their relative rostro-caudal rank: 1, area 10; 2, area 46; 3, area 9/46; 4, Broca's area; 5, Fo; 6, FEF; 7, M-Face; 8, M-Hand. The dashed lines indicate the level of statistical threshold at *p* < 0.001 (Z = ± 0.2).

To compare the connectivity profile of each CMA with the various lateral frontal ROIs, we constructed boxplots corresponding to the correlation strength of each CMA location (RCZa, RCZp, CCZa, and CCZp) with each of the 8 ROIs (Figure 4B). Note that we divided the CCZ into an anterior and posterior part in view of the fact that there were two distinct CCZ hand representations. This allowed us to compare the connectivity profiles of the two CCZ hand representations. The correlation strength of each CMA location with a particular ROI was obtained by averaging the correlation values from its constituent motor representations with the same ROI. The CCZa and CCZp locations contained the anterior and posterior CCZ hand representations respectively. Based on these boxplots, it can be discerned that more frontal CMAs had stronger connectivity with prefrontal regions and weaker connectivity with premotor and motor areas, whereas the opposite was true for more posterior CMAs –reflecting a rostro-caudal coupling of the CMAs with the lateral frontal cortex. The statistical significance of these effects with connectivity *z* values was tested via a general linear model with CMA location (RCZa, RCZp, CCZa, and CCZp), ROI zone (prefrontal and motor cortex) and their interaction as factors. Note that in the second factor, the prefrontal and motor zones were, respectively, obtained by pooling the correlation values of the 5 prefrontal ROIs (a10, a46, a9/46, Broca, Fo) and 3 motor/premotor ROIs (FEF, M-H, and M-T).

We then characterized the rostro-caudal functional axis based on the correlation profiles of the CMAs with the lateral frontal cortex by estimating linear trends in the correlation strength for each CMA location (RCZa, RCZp, CCZa, and CCZp) with the 8 rostro-caudal lateral frontal ROIs. The 8 ROIs were first ranked along a rostro-caudal axis based on their averaged Y coordinate values across subjects and recoded into a numeric axis variable (ROIline): a10 (most anterior)-1, a46 - 2, a9/46 - 3, Broca - 4, Fo - 5, FEF - 6, M-T - 7, M-H (most posterior) - 8. We then performed linear regressions of the connectivity *z* values on CMA location and the linear axis variable (ROIline).

Additionally, we were interested in whether the connectivity profiles of the distinct motor representations (hand, eye, tongue) in the CMAs adhered to the same rostral-caudal functional organization. We focused on the two rostral CMAs (RCZa and RCZp) that both had a complete set of hand, tongue and eye motor representations. Given that the locations of the face motor representations (eye and tongue) in the RCZs varied depending on cingulate sulcal morphology (i.e., whether a PCGS was present), we also questioned if the connectivity profiles of the CMA motor representations were affected by the presence of a PCGS. To test the possible effects of the type of motor representation and cingulate morphology on the rostro-caudal connectivity organization in each RCZ, we linearly regressed the connectivity z values on motor representation type (hand, eye, and tongue), cingulate morphology (with or without PCGS) and the linear axis variable (ROIline). Here, the significance of the motor representation-ROIline and cingulate morphology-ROIline interaction terms would indicate, respectively, if motor representation and cingulate morphology significantly impacted the rostro-caudal connectivity organization in the RCZs.

Importantly, all linear model fits were evaluated using graphic diagnostic tools to ensure that all linear model assumptions were met.

## Results

### Morphological description of the cingulate cortex

Based on individual cingulate morphology, subjects could be divided into 3 main groups (Table 1): Group 1 included 10 subjects who had a PCGS in one hemisphere but not the other. These subjects were further divided into two sub-groups depending on whether a PCGS appeared on the left (Group 1A; 7 subjects) or right hemisphere (Group 1B; 3 subjects). Group 2 consisted of 4 subjects with PCGS on both hemispheres. Lastly, Group 3 consisted of 7 subjects with no PCGS either on the left or the right hemispheres. Note that the CGS and/or the PCGS could be segmented as opposed to a single continuous sulcus. In this case, the number of segments were also reported in Table 1.

**Table 1:** Morphological sulcal variability in the cingulate cortex.

		**Left hemisphere**		**Right hemisphere**	
		**CGS**	**PCGS**	**CGS**	**PCGS**
Group 1A	S2	Not segmented	Not segmented	2 segments	–
	S5	Not segmented	Not segmented	Not segmented	–
	S7	Not segmented	3 segments	2 segments	–
	S8	Not segmented	Not segmented	Not segmented	–
	S11	Not segmented	Not segmented	2 segments	–
	S14	Not segmented	Not segmented	Not segmented	–
	S19	Not segmented	Not segmented	2 segments	–
Group 1B	S13	2 segments	–	Not segmented	Not segmented
	S16	Not segmented	–	2 segments	2 segments
	S20	Not segmented	–	Not segmented	Not segmented
Group 2	S3	Not segmented	Not segmented	Not segmented	Not segmented
	S6	Not segmented	2 segments	2 segments	Not segmented
	S9	2 segments	Not segmented	Not segmented	3 segments
	S17	Not segmented	2 segments	Not segmented	Not segmented
Group 3	S1	2 segments	–	3 segments	–
	S4	Not segmented	–	Not segmented	–
	S10	Not segmented	–	2 segments	–
	S12	Not segmented	–	2 segments	–
	S15	Not segmented	–	2 segments	–
	S18	Not segmented	–	Not segmented	–
	S21	2 segments	–	Not segmented	–

### Task-related fMRI data

#### Activity within the cingulate cortex

The goal of the task-related fMRI study was to map the hand, tongue, and eye motor representations within the various CMAs as seeds for the subsequent resting-state connectivity analyses.

To map the hand representations in the various CMAs, we compared the BOLD signal during right hand movements with the BOLD signal during ocular fixation. Subject-level analysis was performed to assess the impact of the presence of a paracingulate sulcus on the location of this activity. Four hand-motor related activity peaks were observed: (1) at the intersection of the CGS and the vertical paracingulate sulcus (vpcgs) (anterior peak), (2) at the intersection of the CGS and the pre-paracentral sulcus (prepacs) (middle peak), (3) at the intersection of the CGS and the paracentral sulcus (PACS) and (4) posterior to this intersection. Thus, the locations of the two anterior peaks related to the hand fell within the RCZa and RCZp, respectively. The locations of the two posterior peaks related to the hand fell within the CCZ. Indeed, as previously shown (Amiez and Petrides, 2014), the CCZ displays 2 peaks related to hand movement. In the following resting-state data analysis, note that we dissociate the peak located at the intersection of the CGS and the PACS (CCZa) from the peak located posterior to this intersection (CCZp), in an effort to disentangle their relative functional connectivity pattern.

It was also evident that the presence of a PCGS did not have an impact on the location of these hand-related activity peaks in RCZa and RCZp (note that a PCGS is never observed at the level of CCZ). Note that the activity remained in the CGS even when a PCGS was present (with the exception of 2/21 subjects who showed hand motor-related activity in the PCGS in RCZa). These results were in line with our previous findings (Amiez and Petrides, 2014). Finally, we also found that the performance of right handed movements evoked activity in the CMA hand motor representations, bilaterally. Average MNI coordinates of hand movement representations in the cingulate cortex in left and right hemispheres with or without a PCGS are shown in Table 2 and Figure 2.

**Table 2:** Average coordinates of hand, tongue and eye motor representations in RCZa, RCZp, and CCZ in hemispheres in which the PCGS is present vs. absent.

	**NO PCGS**			**Peak location**	**PCGS**			**Peak location**
	**Average MNI coordinates ± sem**				**Average MNI coordinates ± sem**			
	**X**	**Y**	**Z**		**X**	**Y**	**Z**	
**LEFT HEMISPHERE**								
**RCZa**								
Hand	−4.4 ± 0.7	18.1 ± 3.4	39 ± 3	CGS	−6.1 ± 1	15.4 ± 2.8	37.9 ± 1.7	CGS
Tongue	−4.3 ± 0.8	16.2 ± 2.9	34 ± 2.4	CGS	−7.7 ± 0.8	27.7 ± 2.6	36.5 ± 2.1	PCGS
Eye	−7.2 ± 1.6	23 ± 4.2	36 ± 3.5	CGS	−7.6 ± 0.9	31.6 ± 4.2	35.7 ± 3.1	PCGS
**RCZp**								
Hand	−6.1 ± 0.7	0.4 ± 1.5	47.6 ± 1.8	CGS	−5.6 ± 1.3	1.4 ± 1.8	46.3 ± 3.5	CGS
Tongue	−5.5 ± 0.7	1.7 ± 1.1	50.2 ± 2.1	CGS	−5.8 ± 0.3	0.1 ± 1.8	54.8 ± 1.9	PCGS
Eye	−6.8 ± 1	4.8 ± 0.9	49.5 ± 1.6	CGS	−7.3 ± 1.1	6 ± 3.7	50.1 ± 2.5	PCGS
**CCZ**								
Hand ant	−8.2 ± 1.2	−18.7 ± 1.8	48.5 ± 1.1	CGS	−5.9 ± 1	−22 ± 1.7	48.6 ± 1.3	CGS
Hand post	−11 ± 0.7	−27.5 ± 2.6	43.5 ± 2.8	CGS	−7.3 ± 0.8	−33.5 ± 3.5	48.8 ± 2	CGS
**RIGHT HEMISPHERE**								
**RCZa**								
Hand	8.5 ± 1	23 ± 3.1	35 ± 2.4	CGS	9 ± 1.8	26 ± 4.7	28.9 ± 4	CGS
Tongue	9.6 ± 0.9	24.9 ± 2	36 ± 2	CGS	8 ± 1.2	30.3 ± 2.4	38.3 ± 2	PCGS
Eye	9.8 ± 1.1	31.2 ± 5.6	28.8 ± 4.4	CGS	6.6 ± 0.6	39 ± 2.8	38.8 ± 1.9	PCGS
**RCZp**								
Hand	8.1 ± 0.8	2.7 ± 1.8	43.7 ± 1.5	CGS	8 ± 0	−4 ± 2	49 ± 1	CGS
Tongue	8.8 ± 0.9	4.2 ± 2.4	41.1 ± 2.3	CGS	7.7 ± 1.5	2.3 ± 4.9	53.3 ± 1.8	PCGS
Eye	7.4 ± 0.8	8.4 ± 2.8	42.9 ± 2.7	CGS	11 ± 1.5	9.6 ± 2.4	48.2 ± 3.8	PCGS
**CCZ**								
Hand ant	11 ± 1.1	−24.4 ± 1.7	43.2 ± 1.4	CGS	9.5 ± 1.8	−18 ± 1.8	44.8 ± 1.1	CGS
Hand post	9.9 ± 1.6	−35 ± 1.3	49.6 ± 1.1	CGS	11.5 ± 6.5	−33.5 ± 5.5	45 ± 5	CGS

To map the tongue movement representations in the various CMAs, we contrasted the BOLD signal during tongue movements with the BOLD signal during ocular fixation. Tongue-motor related activity was observed: (1) at the intersection of the CGS and the vpcgs when no PCGS was present, and at the intersection of the PCGS and the vpcgs when a PCGS was present (anterior peak), (2) at the intersection of the CGS and prepacs when no PCGS was present and at the intersection of the PCGS and prepacs when a PCGS was present (middle peak). These observations suggested that the anterior and the middle peaks were located in the RCZa and RCZp, respectively. Note that both peak locations were clearly influenced by the presence of a PCGS: when a PCGS was present, tongue-motor related activity was found in the PCGS (with the exception of 2/21 subjects who had tongue motor activity in the CGS in RCZa). The absence of a third posterior peak corroborated our earlier observation that the CCZ does not contain tongue movements representation. These results are congruent with our previous observations (Amiez and Petrides, 2014). Finally, as with hand movements, tongue movements evoked bilateral activation in the CMA tongue regions. The average MNI coordinates of tongue movement representations in the cingulate cortex in left and right hemispheres with and without a PCGS are in Table 2 and Figure 2.

To map the saccadic eye movement representations in the various CMAs, we compared the BOLD signal during saccadic eye movements with that during ocular fixation. Saccadic eye movement-related activity was observed: (1) at the intersection of the CGS and the vPCGS when no PCGS was present and at the intersection of the PCGS and the vPCGS when it was present (anterior peak), (2) at the intersection of the CGS and prepacs when no PCGS was present and at the intersection of the PCGS and prepacs when it was present (middle peak). These observations suggest that the anterior and the middle peaks belong, respectively, to RCZa and RCZp. The location of the two peaks were impacted by the presence of PCGS: both saccadic movement-related activity peaks were located in the PCGS when it was present (with the exception of 1/21 subjects which showed saccadic eye movement activity in the CGS in RCZp). As with tongue movements, a third posterior saccadic movement related peak was absent confirming that there is no saccadic eye movement representation in the CCZ. The above results were in line with our previous observations (Amiez and Petrides, 2014). Finally, we also observed that saccadic eye movements induced bilateral activation in the CMAs. The average MNI coordinates of saccadic eye movement representations in the cingulate cortex in left and right hemispheres with and without a PCGS are shown in Table 2 and Figure 2.

Across all subjects, the 8 motor-related peaks could be observed in the cingulate cortex (3 peaks in RCZa, 3 in RCZp, and 2 in CCZ) in both hemispheres. Note that not all subjects showed all of the 8 peaks. On average, subjects display 6.1 peaks ± 1.2 stdev in the left hemisphere and 5.1 peaks ± 1.7 stdev in the right hemisphere, consistently with the results of Amiez and Petrides (2014).

#### Activity within the precentral gyrus motor cortex

We used the same contrasts described above to identify the primary hand motor region (hand vs. fixation), primary tongue motor region (tongue vs. fixation), and FEF (saccades vs. fixation). Subject by subject analysis confirmed, for each subject, the location of the hand motor region in the precentral knob in the central sulcus, the location of the tongue motor region in the posterior part of the ventral precentral gyrus, and the location of the FEF in the ventral branch of the superior precentral sulcus. The average x, y, z coordinates ± s.e.m. of precentral motor ROIs in each of the 21 subjects are presented in Table 3.

**Table 3:** Average coordinates of hand, tongue and eye motor representations in motor areas.

	**MNI coordinates ± sem**			**Location**
	**X**	**Y**	**Z**	
**LEFT HEMISPHERE**				
Hand motor area	−33.9 ± 1.02	−26.3 ± 0.58	58.2 ± 0.99	Precentral knob in the central sulcus
Tongue motor area	−55.9 ± 1.04	−4.6 ± 1.02	32.2 ± 1.14	Ventral precentral gyrus
FEF	−31.7 ± 1.95	−5.8 ± 0.91	53.4 ± 1.22	Ventral branch of the superior precentral sulcus
**RIGHT HEMISPHERE**				
Hand motor area	−	−	−	–
Tongue motor area	58.4 ± 0.92	−2.2 ± 0.90	30.8 ± 1.42	Ventral precentral gyrus
FEF	31 ± 1.93	−2.8 ± 0.98	55.5 ± 1.28	Ventral branch of the superior precentral sulcus

#### Resting state fMRI data

To demonstrate the pattern of intra-hemispheric connectivity of RCZa, RCZp, CCZa, and CCZp seeds with the lateral prefrontal and motor ROIs, we first performed a hierarchical clustering of the seeds and ROIs based on their inter-correlations across all subjects (see Method). Resulting dendrograms from the seed and ROI clustering are displayed in Figure 4A along with a heatmap reflecting the correlation strength between each pair of seed-ROI clusters. This analysis demonstrated the existence of three CMA clusters based on their functional connectivity with the prefrontal and the motor cortex: RCZa, RCZp, and CCZ.

The Boxplots in Figure 4B further depict the average *Z* values of correlations between seeds and ROIs across subjects and, therefore, how the activity of each CMA is differentially correlated with the activity of the prefrontal/motor ROIs. We tested these differences in connectivity z values with a generalized linear model with CMA location (RCZa, RCZp, CCZa, and CCZp) and ROI zones (prefrontal zones: a10, a46, a9/46, Broca, and Fo, and motor zones: FEF, Face –M-T– and Hand –M-H–) as fixed effect. The results indicated that the connectivity profiles with the prefrontal and motor zones differ significantly between CMA (ANOVA, ROIs × CMAs, *df* = 7, *F* = 21.296, *p* < 2 × 10^−16^): (1) the activity of RCZa is more correlated with that of the prefrontal zone but less correlated with that of the motor areas than the RCZp, CCZa, and CCZp, and (2) the activity of RCZp, CCZa, and CCZp are more correlated with that of the motor areas, but less correlated with that of prefrontal areas in comparison with RCZa. This analysis thus further suggests a topological organization of the correlation profiles of the CMAs with lateral frontal areas. We also assessed whether this correlation profiles can be a function of physical distance between seeds and ROIs. We calculated the Euclidian distances between the different seeds and the ROIs tested (Figure 4C). Results strongly suggest that the z-scores (displayed in Figure 4A) do not strictly varies as a function of distance as the shorter distances are not systemically associated with the higher z-scores and vice versa.

Nevertheless, the above analyses do not provide any estimation of the linearity of trends of correlation with lateral frontal areas along the rostro-caudal axis. To test and quantify these linear trends from anterior prefrontal to motor areas, we recoded the various lateral frontal ROIs into a numeric axis variable (ROIline) that corresponded to their relative posterior-to-anterior positions (see Method). Based on this coding, the lowest value (1) corresponds to a10 (the most anterior ROI) and the largest value (8) corresponds to M-H (the most posterior ROI). We then performed multiple linear regressions on the correlation values with CMA location and ROIline as predictors. The analysis revealed a significant CMA x ROIline interaction (Figure 4D, *df* = 3, *F* = 66.3, *p* < 2 × 10^−16^, ANOVA with CMAs and ROIline as factors), which indicates that the linear trends in connectivity within the rostral-to-caudal axis of lateral frontal cortex differed between the CMAs: a negative linear trend was observed for RCZa (more strongly correlated with rostral prefrontal areas), positive slopes were observed for the other CMAs (more strongly correlated with posterior motor areas). Thus, the connectivity profiles of CMAs with the lateral frontal cortex regions follow a rostro-caudal organization: the anterior CMA has stronger functional coupling with the prefrontal cortex and weaker with the motor cortex; the posterior CMAs have stronger coupling with the motor cortex and weaker with the prefrontal cortex.

**Figure 5:**
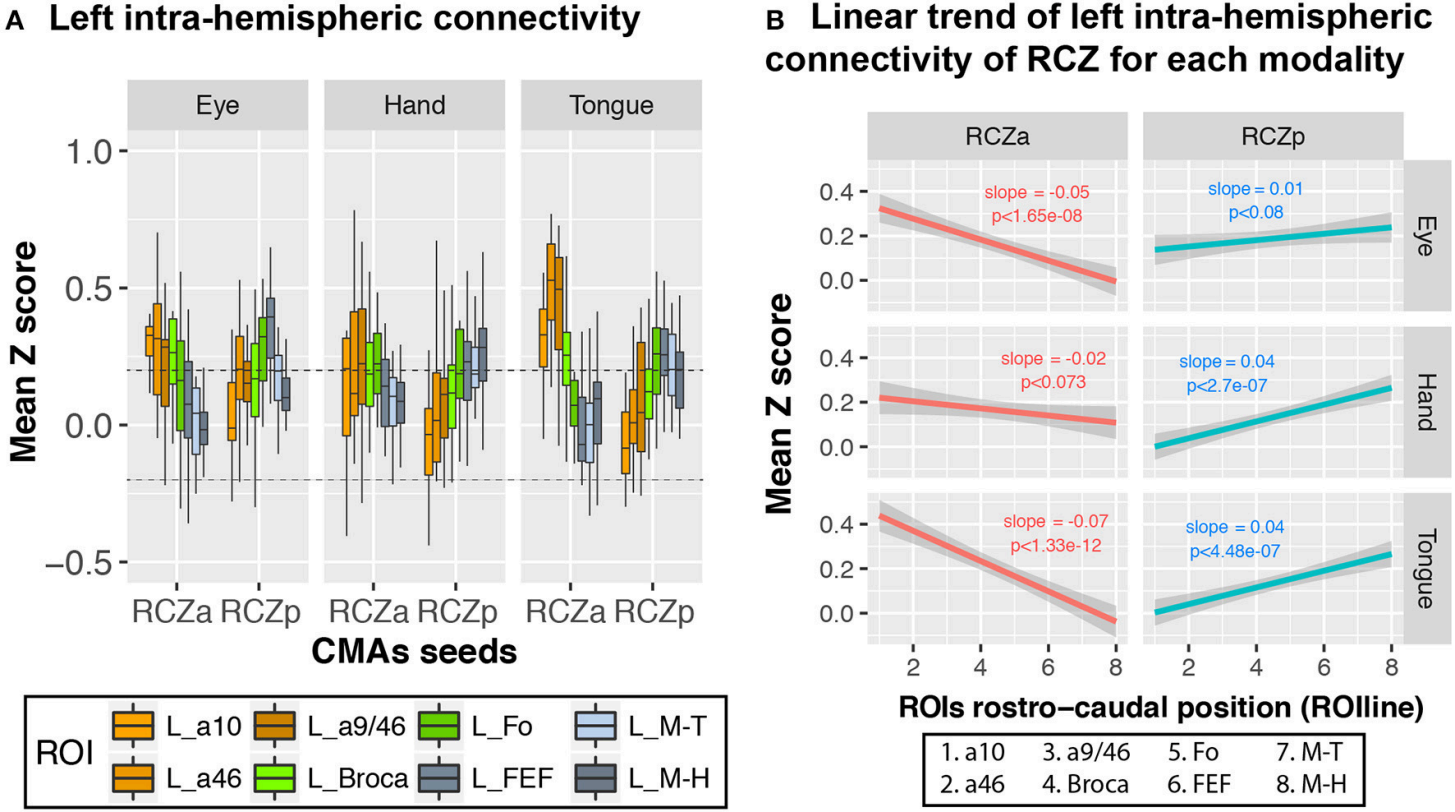
CMA motor representations display linear rostro-caudal organization in connectivity with the lateral frontal cortex ROIs. **(A)** Boxplots displaying the mean (± SD) z-transformed connectivity between each CMA motor representation (eye, hand, tongue) in the RCZs with the various ROIs in all subjects. **(B)** Significant linear rostro-caudal organization in the connectivity of each CMA map with the rostral-caudal lateral frontal ROIs. The rostro-caudal organization variable (ROIline) was obtained by recoding the ROIs in terms of their relative rostro-caudal rank: 1, area 10; 2, area 46; 3, area 9/46; 4, Broca's area; 5, Fo; 6, FEF; 7, M-Face; 8, M-Hand. The dashed lines indicate the level of statistical threshold at *p* < 0.001 (*Z* = ± 0.2).

We further investigated whether the same rostro-caudal cognitive-to-motor pattern of connectivity existed for each motor representation in the CMAs. Because only RCZa and RCZp contain both hand and face (tongue and saccadic eye) movement representations, this analysis was performed only with these two CMAs, independently. Seeds for resting-state data analyses were derived from the CMA motor activation peaks described above, and they are named “motor representations” in the description below. Overall, the rostro-caudal anatomo-functional organization described above is observed for all motor representations in both the RCZa and RCZp (Figure 5A) (Hand motor representation: *df* = 7, *F* = 3.6, *p* < 0.001; Tongue motor representation: *df* = 7, *F* = 17.436, *p* < 2.2 × 10^−16^; Eye motor representation: *df* = 7, *F* = 6.1, *p* < 1.6 × 10^−6^, ANOVA with CMAs type (i.e., RCZa and RCZp) and ROIs as factors). The slopes of seed-ROI connectivity strength against ROIline slightly varied between seed motor representations in RCZa (after stepwise model selection: ROIline × Seed interactions, *df* = 2, *F* = 10.2, *p* < 1 × 10^−4^, ANOVA). This is obviously related to the specific connections between each motor representations with the corresponding primary motor fields (see Figure 5A). Concerning RCZp, these slopes varied marginally (after stepwise model selection: ROIline x Seed interactions, *df* = 2, *F* = 3.02, *p* < 0.05, ANOVA) but they were all negative for RCZa (stronger connectivity with more rostral lateral regions) and all positive for RCZp (stronger connectivity with more caudal regions) (Figure 5B).

**Figure 6:**
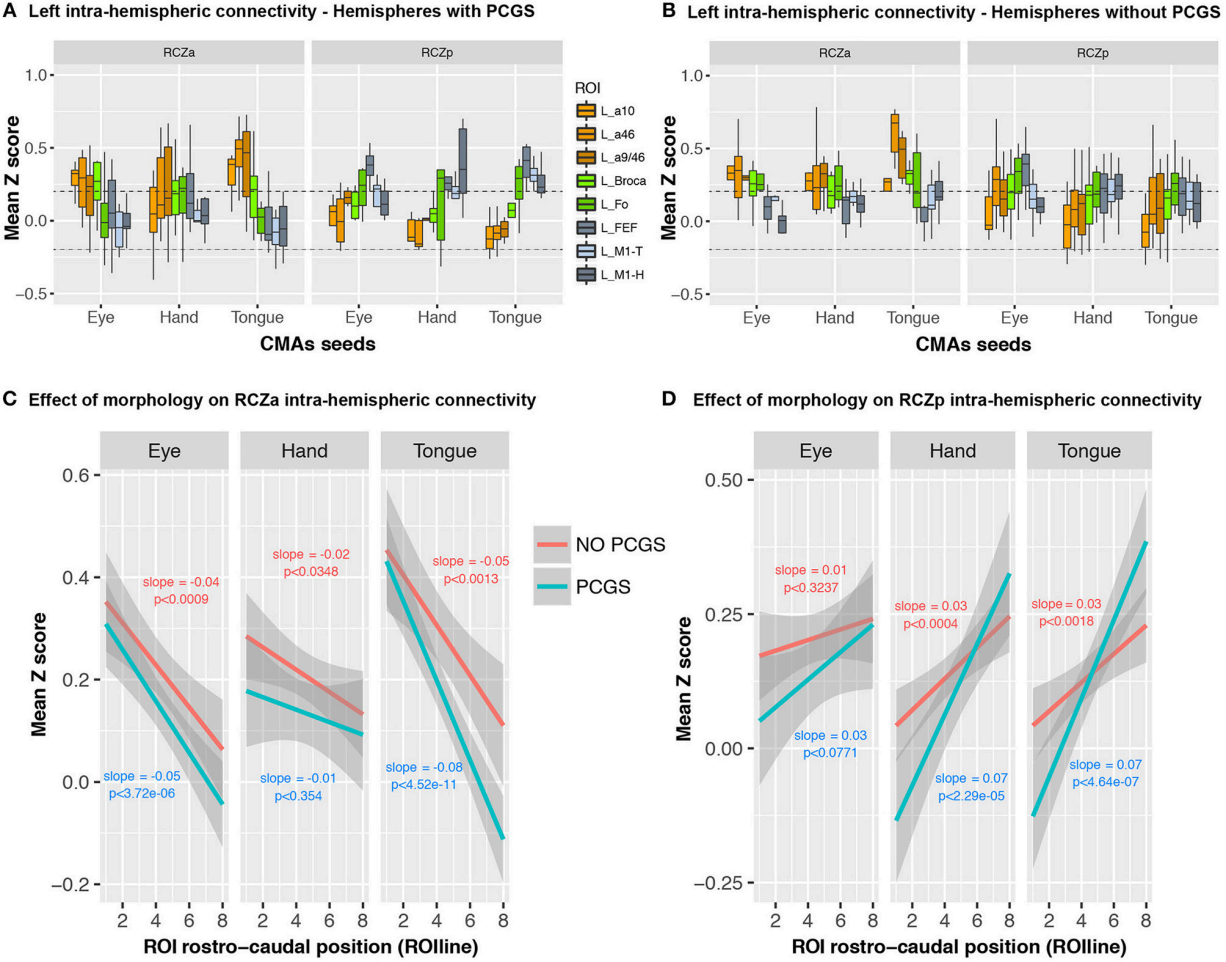
No effect of cingulate morphology on linear rostro-caudal organization in connectivity between CMA motor representations and the lateral frontal cortex ROIs. Boxplots displaying the mean (±SD) z-transformed connectivity between each CMA motor representation (eye, hand, tongue) in the RCZs with the various ROIs are plotted separately for hemispheres with **(A)** and without PCGS **(B)**. Linear trends in connectivity strength are present for all CMA representations, regardless of cingulate morphology, in both RCZa **(C)** and RCzp **(D)**. The dashed lines indicate the level of statistical threshold at *p* < 0.001 (*Z* = ± 0.2).

We then assessed whether cingulate morphology (i.e., the presence or absence of a PCGS, see Table 1) affected the connectivity profiles of RCZa and RCZp. Note that a PCGS is never observed at the level of CCZ. The results show that the rostro-caudal anatomo-functional organization is similar for all motor representations in both the RCZa and RCZp independently of the presence of a PCGS (Figure 6A) (In hemispheres with PCGS: interaction between CMAs identity and ROIs: *df* = 7, *F* = 13.77, *p* < 2 × 10^−16^ but no interaction between CMAs identity, ROIs, and motor representations: *df* = 14, *F* = 1.63, *p* < 0.07; In hemispheres with no PCGS: interaction between CMAs identity and ROIs: *df* = 7, *F* = 7.96, *p* < 4.75 × 10^−9^ but no interaction between CMAs identity, ROIs, and motor representations: *df* = 14, *F* = 0.62, *p* < 0.84, ANOVA). Figure 6B shows that when morphology (presence/absence of a PCGS) is taken into account, connectivity with lateral frontal areas always follows negative and positive slopes for RCZa and RCZp seeds, respectively (Figures 6C,D). In RCZa, these slopes were not influenced by the presence of a PCGS (interaction dropped by model selection) although the main effect of morphology was significant (*df* = 1, *F* = 15.6, *p* < 1 × 10^−4^, ANOVA) showing that overall correlation values (Z) between RCZa and lateral frontal regions were lower in the presence of a PCGS. In RCZp, the morphology did interact with the rostro-caudal organization (interaction after stepwise selection, RCZp, *df* = 2, *F* = 11.87, p < 1 × 10^−3^, ANOVA) as slopes of ROIline for Hand and Tongue representations were steeper than the gradient for Hand representation in presence of a PCGS. Yet, the rostro-caudal slopes of ROIline was positive in all cases in RCZp.

Taken together, these findings suggest that the type of motor representation and cingulate morphology had no qualitative impact on the rostral-caudal functional connectivity organization between the CMAs and the lateral frontal cortex.

## Discussion

The main aim of the present study was to examine the resting-state functional connectivity of the 3 human cingulate motor areas and their motor representations with the lateral prefrontal and motor cortical regions taking into account inter-individual anatomical variations. The present study confirmed the existence of 3 CMAs and their somatotopic organization, as reported in our former study (Amiez and Petrides, 2014) and demonstrated, for the first time, the functional connectivity profiles of the various motor subdivisions within the different CMAs. A key finding that emerged from the present study is that the anterior CMA displays an opposing linear trend of connectivity with the lateral frontal cortex in comparison with the more caudal CMAs. The anterior CMA shows a negative trend of decreasing connectivity strength with rostral-to-caudal lateral frontal regions, whereas positive trends of increasing connectivity strengths with rostral-to-caudal lateral frontal regions are observed for the caudal CMAs.

As in our previous fMRI work which sought to identify the locations of the human CMAs (Amiez and Petrides, 2014), we identified the presence of 3 CMAs that can be predicted from local morphology: the rostral anterior cingulate motor zone (RCZa) is located at the intersection of the CGS or PCGS with the posterior vertical paracingulate sulcus (vPCGS-p), the rostral posterior cingulate motor zone (RCZp) is located at the intersection of the CGS or PCGS with the preparacentral sulcus (prepacs), and the CCZ is located at the intersection of the CGS and the paracentral sulcus (pacs) and extends posterior to this intersection. Importantly, the present data confirmed that the hand motor representation in RCZa and RCZp is located in the CGS independently of the presence of a PCGS. By contrast, the face motor representation in both these cingulate motor areas is located in the CGS when the PCGS is absent, but in the PCGS when present. The location of the monkey homologs of the human CMAs remains debated. However, Picard and Strick (1996, 2001, 2003) and then Amiez and Petrides (2014) suggested that the CMAr, CMAv, and CMAd in the monkey may be homologous to the human RCZa, RCZp, and CCZ motor zones, respectively. These suggested homologies are based on several pieces of evidence: (1) Intracortical microstimulations in the macaque cingulate cortex has shown that CMAr contains a face, a hand, and a leg representation, and that CMAd contains 2 arm representations and a leg representation. Correspondingly, the same sets of motor maps have been observed in the human RCZa (one face, hand and leg representation) and CCZ (two hand and one leg representation) (Amiez and Petrides, 2014). In the CMAv, microstimulation studies have shown a hand and a leg but not a face motor representation. However, a connectivity study (Wang et al., 2004) and a (14C)-2-deoxyglucose functional imaging study (Moschovakis et al., 2004) in the monkey reported the existence of a cingulate eye field located adjacent to the forelimb movement region within the CMAv, strongly suggesting that CMAv might contain a face motor representation. This point has recently been confirmed in a macaque neuroimaging study (Cléry et al., 2018). CMAv could therefore be considered the monkey homolog of the human RCZp, since both display a hand, a leg, and a face motor representation.

The connectivity profiles of the 3 human CMAs (RCZa, RCZp, and CCZ) with lateral frontal regions reflect a linear rostro-caudal organization. The anterior CMA's activity is strongly correlated with that of the prefrontal cortex, and weakly correlated with that of the motor cortex, exhibiting a negative rostro-caudal linear trend of correlation strength. In contrast, the activity of posterior CMAs (RCZp and CCZ) is strongly correlated with that of the motor cortex, and weakly correlated with that of the prefrontal cortex. These results are consistent with experimental anatomical data from non-human primate studies (Dum and Strick, 1991; Bates and Goldman-Rakic, 1993; He et al., 1995; Petrides and Pandya, 1999, 2002; Wang et al., 2001, 2004) and support the functional homology between human and monkey CMAs. Although all CMAs contain projections to the motor cortex and spinal cord, the connections are progressively denser as one proceeds from the rostral to the caudal CMAs: CMAr, CMAd, and finally CMAv (Dum and Strick, 1991; Bates and Goldman-Rakic, 1993; He et al., 1995; Wang et al., 2001, 2004). This organization suggests an increased role of caudal CMAs in motor control. In addition, micro-stimulations in caudal, as compared to rostral, CMAs evoked movements more consistently and with reduced onset latencies (Morecraft and Tanji, 2009). In contrast, interconnections with the prefrontal cortex are more commonly present in rostral CMA (Bates and Goldman-Rakic, 1993; Lu et al., 1994), suggesting an increased role in higher-order cognitive processing compared with posterior CMAs. Furthermore, monkey CMAr also receives denser dopaminergic projections from the ventral tegmental area (Williams and Goldman-Rakic, 1998), supporting its involvement in feedback-driven behaviors (Quilodran et al., 2008). It should also be noted that the rostral, but not the caudal, CMAs have connections with vocalization-related brain regions (Vogt and Barbas, 1988; An et al., 1998) and also the ventrolateral prefrontal cortical area 45 (Petrides and Pandya, 2002), consistent with their potential role in voluntary vocal and orofacial control (Loh et al., 2016). Given the anatomical-functional correspondence between the human and macaque CMAs (Picard and Strick, 2001; Amiez and Petrides, 2014), one could expect the functional organization of the CMAs to be similar between the two species. The present results provide strong support for this hypothesis.

The connectivity profiles of the 3 CMAs with Fo demonstrate major differences: Fo displays positive correlations with all motor representations of RCZp, but only with the hand motor representation of RCZa. By contrast, CCZ is not functionally connected with Fo (Figure 4B). Although the literature shows that the anterior MCC -where the RCZa probably lies (Procyk et al., 2016)- is systematically co-activated with Fo in fMRI studies assessing performance monitoring abilities (e.g., Amiez et al., 2012a,b, 2013, 2016), the functional relationships between the two structures and the role of Fo are not understood. The present results strongly suggest a complex relationship between the anterior MCC and Fo and future studies are required to disentangle the role of Fo in performance monitoring.

Importantly, the demonstration that the CMAs follow a rostral-to-caudal organization in their links with lateral frontal cortex also contributes to the growing body of evidence that the human frontal cortex is organized along a rostro-caudal axis (Petrides, 2005a,b; Koechlin and Summerfield, 2007; Badre and D'Esposito, 2009) with parallel links to the medial cingulate motor region. Several hypotheses suggest that progressively rostral lateral frontal regions are linked with increasingly complex and abstract processes and rules for behavioral control. For instance, Petrides has provided evidence that the mid-dorsolateral prefrontal region is critical for the monitoring of information in working memory (the epoptic process) while more posterior lateral frontal regions are involved in the allocation of attention to environmental stimuli and movement selection (Petrides, 2005a,b, 2013). A few studies have shown that more rostral/caudal CMAs could be involved in monitoring the reliability of higher/lower-order behavioral actions and rules, and initiating searches for alterative options as existing ones become obsolete (Kouneiher et al., 2009; Domenech and Koechlin, 2015). Congruent with these propositions, Stoll et al. (2016) found increased neuronal activity in the rostral mid-cingulate cortex for decisions to shift away from a default task. Regarding caudal regions, Debaere et al. (2003) found that performing coordinated hand movements with external visual guidance (when a simple behavioral rule, i.e., performing action according to visual cue, is valid) vs. without (when behavioral rule is invalid and there is a need to self-initiate novel actions) activated, respectively, the caudal lateral prefrontal vs. the caudal MCC.

The present study revealed, for the first time, the functional connectivity profiles of the various CMA motor representations with the lateral prefrontal and motor areas. We found that the connectivity profiles of CMA motor representations are similar within the same CMA. For instance, the connectivity profile of the RCZa face representation is more similar to that of the RCZa hand representation than the RCZp face representation. This strongly indicates that the motor representations found in a particular CMA likely function as part of the same CMA. Also, RCZa and RCZp face motor representations that were situated in the paracingulate sulcus or the cingulate sulcus (when the paracingulate sulcus was absent) exhibited similar connectivity trends. This finding indicates that even though the presence of a paracingulate sulcus influences the physical location of the face motor representations in RCZa and RCZp, their functional position remains the same. Likewise, the trends in the connectivity patterns of the hand motor representations are also conserved regardless of the presence of a paracingulate sulcus.

To the best of our knowledge, only two previous studies had sought to characterize the connectivity profile of human CMAs, one via diffusion-tract imaging (DTI; Beckmann et al., 2009) and the other via resting-state fMRI (Habas, 2010). In the study by Beckmann et al. (2009), three seed clusters corresponding to the three CMAs of the human brain were obtained via blind connectivity-based parcellations performed on voxels within the cingulate cortex. The most rostral cluster 4 (corresponding to RCZa) showed the highest connection probability with the dorsal prefrontal cortex, while their most caudal cluster 6 (CCZ) had the highest connection probability with the parietal and motor cortex (Beckmann et al., 2009). Habas (2010) examined the resting-state functional connectivity of the rostral cingulate sulcal region extending 10 mm anterior to the vertical line from the anterior commissure and a caudal cingulate sulcal region extending 10 mm posterior to the anterior commissure and showed that the rostral region had higher connectivity with prefrontal and language-associated cortical areas while the caudal region had increased connectivity with sensory-motor regions. Notably, both studies had shown that, as in the macaque, the human CMAs generally exhibit a rostro-caudal functional organization with progressively rostral/caudal CMAs being associated with more cognitive/motor brain regions. However, there are two important limitations associated with the above studies: in both investigations, the CMA seeds had not been defined on the basis of task-based fMRI, and not on individual subject brain morphology. The present study provides new insights into the functional organization of the human CMAs and, crucially, their motor representations and their relationship with lateral frontal areas.

Note that a limitation of the present study is that the selection of the ROIs in the prefrontal cortex has been based of their known frequent co-activation with the MCC in a large range of cognitive tasks (Amiez and Petrides, 2007; Amiez et al., 2012b, 2013). The extent of functional connectivity of the CCZ with the prefrontal cortex may therefore have been underestimated. However, at the present time of knowledge, studies aiming to assess the putative role of the CCZ are critically lacking. Only one recent study has shown the existence of a cingulate sulcus visual area (CSv) at the level of CCZ. This region is anatomically and functionally connected with the ventral premotor area 6 and sensory areas involved in processing moving visual (V6) and vestibular (VIP) stimuli, suggesting a role in the online control of locomotion (Smith et al., 2017). The authors report also a lack of connections with their ventrolateral prefrontal cortical ROIs (areas 44, 45, and 47). Our results are consistent with these findings, suggesting that this region -occupied by CCZ and CSv- is functionally connected with the motor system but not with the prefrontal cortex. Future studies should focus on (1) how the CSv relates to the two hand representations in the CCZ, and (2) how these areas are anatomically and functionally connected to the whole prefrontal cortex.

To conclude, the present study demonstrated that the 3 CMAs are organized along a rostro-caudal organization in parallel with the prefrontal and motor areas and could likely contribute to different hierarchies of cognitive-motor controls.

## Author contributions

KL set up the experiment, acquired data, analyzed the task-related fMRI data, and wrote the paper. FH-B analyzed the resting state data, and wrote the paper. MP wrote the paper. EP performed the statistics and wrote the paper. CA set up the experiment, analyzed the resting state data, and wrote the paper.

### Conflict of interest statement

The authors declare that the research was conducted in the absence of any commercial or financial relationships that could be construed as a potential conflict of interest.
